# Reply to “Comment on: Effects of Nigella Sativa on Type-2 Diabetes Mellitus: A Systematic Review”

**DOI:** 10.3390/ijerph17093111

**Published:** 2020-04-29

**Authors:** Amiza Hamdan, Ruszymah Haji Idrus, Mohd Helmy Mokhtar

**Affiliations:** Department of Physiology, Faculty of Medicine, Universiti Kebangsaan Malaysia, Kuala Lumpur 56000, Malaysia; amizahamdan@gmail.com (A.H.); ruszyidrus@gmail.com (R.H.I.)

Thank you for your interest and the comments [1] on our review article “Effects of Nigella Sativa on Type-2 Diabetes Mellitus: A Systematic Review” [2]; we believe research into natural products needs to be supported, as they are at the forefront of drug discovery.

According to the guidelines, at least two databases were required to retrieve all included studies [3]. In this review, two databases, Scopus and Medline via Ebscohost, have been used to identify relevant studies about the reported effect of Nigella sativa (NS) on type-2 diabetes mellitus (T2DM), which in line with the guidelines.

We also had restricted the selection of articles to only original articles that were published in English in order to avoid misinterpretation of non-English language articles. The guidelines on including articles in languages other than English are still lacking. In addition, it is costly and time-consuming to include non-English articles due to the lack of language resources to translate these articles [4].

Most aspects of this review are reported according to the Preferred Reporting Items for Systematic Reviews and Meta-Analysis (PRISMA) statement guidelines. However, the protocol of this review is not registered in the database of systematic review protocols.

Finally, thank you for the comments and we will take note for our future reviews.
